# (*E*)-1-([1,1′-Biphen­yl]-4-yl)-3-(2-methyl­phen­yl)prop-2-en-1-one

**DOI:** 10.1107/S1600536814014317

**Published:** 2014-06-25

**Authors:** D. Shanthi, T. Vidhya Sagar, M. Kayalvizhi, G. Vasuki, A. Thiruvalluvar

**Affiliations:** a Department of Chemistry, Annamalai University, Annamalai Nagar 608 002, Tamilnadu, India; b Department of Physics, Kunthavai Naachiar Government Arts College (W) (Autonomous), Thanjavur 613 007, Tamilnadu, India; c Postgraduate Research Department of Physics, Rajah Serfoji Government College (Autonomous), Thanjavur 613 005, Tamilnadu, India

**Keywords:** crystal structure

## Abstract

In the title mol­ecule, C_22_H_18_O, the *o*-tolyl ring is connected through a conjugated double bond. The mol­ecule adopts an *E* conformation and the C—C=C—C torsion angle is 178.77 (13)°. The overall conformation may be described by the values of dihedral angles between the different planes. The terminal rings are twisted by an angle of 54.75 (8)°, while the biphenyl part is not planar, the dihedral angle between the planes of the rings being 40.65 (8)°. The dihedral angle between the benzene rings is 14.10 (7)°. There are three weak C—H⋯π inter­actions found in the crystal structure. No classic hydrogen bonds are observed.

## Related literature   

For the bioactivity of chalcones, see: Dimmock *et al.* (1999 ▶). For biological applications of chalcones, see: Opletalova (2000 ▶); Opletalova & Sedivy (1999 ▶). For chalcones as non-linear optical materials, see: Fichou *et al.* (1988 ▶); Goto *et al.* (1991 ▶). For further applications of chalcones, see: Sarojini *et al.* (2006 ▶). For the crystal structures of related compounds, see: Betz *et al.* (2011*a* ▶,*b* ▶). For bond-length data, see: Allen *et al.* (1987 ▶).
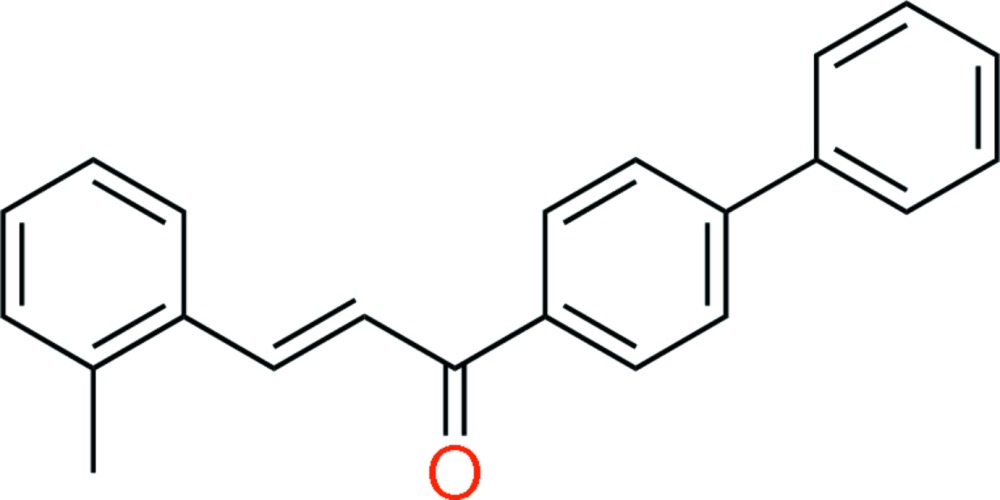


## Experimental   

### 

#### Crystal data  

C_22_H_18_O
*M* *_r_* = 298.36
Triclinic, 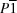

*a* = 7.6396 (3) Å
*b* = 9.9106 (4) Å
*c* = 11.9263 (4) Å
α = 103.166 (2)°
β = 104.713 (2)°
γ = 103.308 (2)°
*V* = 809.66 (6) Å^3^
*Z* = 2
Mo *K*α radiation
μ = 0.07 mm^−1^
*T* = 273 K
0.40 × 0.35 × 0.30 mm

#### Data collection  

Bruker Kappa APEXII CCD diffractometer
Absorption correction: multi-scan (*SADABS*; Bruker, 2004 ▶) *T*_min_ = 0.908, *T*_max_ = 1.000
18636 measured reflections
4534 independent reflections
3291 reflections with *I* > 2σ(*I*)
*R*_int_ = 0.024

#### Refinement  

*R*[*F* ^2^ > 2σ(*F* ^2^)] = 0.048
*wR*(*F* ^2^) = 0.151
*S* = 1.06
4534 reflections
209 parameters
H-atom parameters constrained
Δρ_max_ = 0.23 e Å^−3^
Δρ_min_ = −0.17 e Å^−3^

### 

Data collection: *APEX2* (Bruker, 2004 ▶); cell refinement: *APEX2* and *SAINT* (Bruker, 2004 ▶); data reduction: *SAINT* and *XPREP* (Bruker, 2004 ▶); program(s) used to solve structure: *SHELXS2013* (Sheldrick, 2008 ▶); program(s) used to refine structure: *SHELXL2014* (Sheldrick, 2008 ▶); molecular graphics: *ORTEP-3 for Windows* (Farrugia, 2012 ▶) and *PLATON* (Spek, 2009 ▶); software used to prepare material for publication: *SHELXL2014* and *PLATON* (Spek, 2009 ▶).

## Supplementary Material

Crystal structure: contains datablock(s) global, I. DOI: 10.1107/S1600536814014317/jj2188sup1.cif

Structure factors: contains datablock(s) I. DOI: 10.1107/S1600536814014317/jj2188Isup2.hkl

Supporting information file. DOI: 10.1107/S1600536814014317/jj2188Isup3.cdx

Supporting information file. DOI: 10.1107/S1600536814014317/jj2188Isup4.cml

CCDC reference: 977614

Additional supporting information:  crystallographic information; 3D view; checkCIF report

## Figures and Tables

**Table 1:** Hydrogen-bond geometry (Å, °) *Cg*1, *Cg*2 and *Cg*3 are the centroids of the C2–C7 methyl­benzene, C11–C16 benzene and C17–C22 phenyl rings, respectively.

*D*—H⋯*A*	*D*—H	H⋯*A*	*D*⋯*A*	*D*—H⋯*A*
C1—H1*C*⋯*Cg*2^i^	0.96	2.97	3.6689 (17)	131
C5—H5⋯*Cg*3^ii^	0.93	2.84	3.5126 (18)	130
C21—H21⋯*Cg*1^iii^	0.93	2.99	3.632 (2)	127

Symmetry codes: (i) 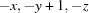; (ii) 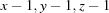; (iii) 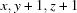.

**Fig. 1.**
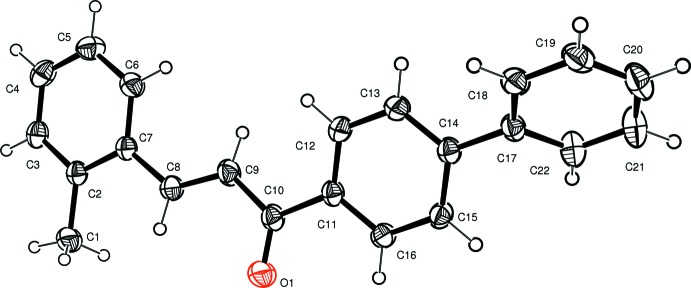
The molecular structure of the title compound, with displacement ellipsoids drawn at the 30% probability level. H atoms are shown as small spheres of arbitrary radius.

**Fig. 2.**
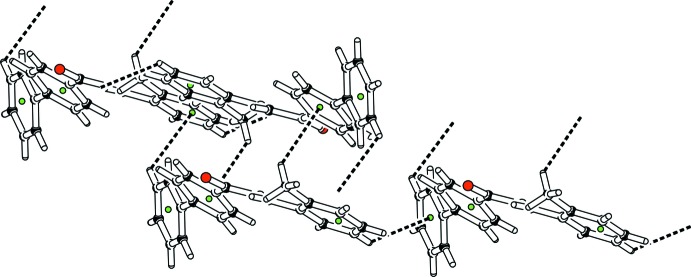
The partial packing of the title compound, showing the three weak C—H···π interactions.

## supplementary crystallographic information

### S1. Comment

Bioactivities of chalcones were reported by Dimmock *et al.*, (1999). The antibacterial, fungistatic and fungicidal properties of these compounds have also been reviewed (Opletalova *et al.* 2000, 1999). In addition with appropriate substituents, chalcones are a class of non-linear optical materials (Fichou *et al.* 1988, Goto *et al.* 1991). Recently, it has been noted that among many organic second harmonic generation, chalcone derivatives have excellent blue light transmittance and good cyrstallizability (Sarojini *et al.* 2006). The related compounds whose structures have been solved by X-ray are (2E)-1-(4,4''-Difluoro-5'-methoxy-1,1':3',1''-terphenyl-4'-yl)- 3-(4-fluorophenyl)prop-2-en-1-one (Betz *et al.* 2011*a*) and (*E*)-1-(4,4''-Difluoro-5'-methoxy-1,1':3',1''-terphenyl-4' -yl)-3-(4-nitrophenyl)prop-2-en-1-one (Betz *et al.* 2011*b*).

In the title molecule (Fig. 1), C_22_H_18_O, the *o*-tolyl ring is connected through a conjugated double bond. The molecule adopts an E configuration and the C7—C8—C9—C10 torsion angle is 178.77 (13)°. Further, the torsion angle [C8—C9—C10—C11 = -164.91 (13)°] shows that the prop-2-en-1-one unit is not planar. The overall conformation of the compound may be described by the values of dihedral angles between the different planes. The terminal rings (C2—C7) and (C17—C22) are twisted by an angle of 54.75 (8)°, while the biphenyl part is not planar, the dihedral angle between the planes of the rings (C11—C16) and (C17—C22) being 40.65 (8)°. The dihedral angle between the benzene rings (C2—C7) and (C11—C16) is 14.10 (7)°.

There are three weak C1—H1C···π, C5—H5···π and C21—H21···π interactions involving the central benzene ring (C11—C16), the terminal phenyl ring (C17—C22) and the terminal benzene ring (C2—C7), respectively, are found in the crystal structure. The C_ar_—C*sp*^3^, C_ar_—C_ar_ and C═O bond lengths in (I) are within their normal ranges (Allen *et al.*, 1987). No classic hydrogen bonds are observed.

### S2. Experimental

Biphenyl acetone (1.06 g, 10 mmol) and 2-methylbenzaldehyde (1.06 g, 10 mmol) in ethanol (25 ml) is mixed in the presence of NaOH (10 ml 30%). The reaction mixture was stirred for 6 h. Then the contents of the flask were poured into ice cold water (250 ml) and left for 12 h. The solid obtained was filtered and recrystallized for three to four times with ethanol. The colourless single crystals of the title compound used for X-ray diffraction studies were grown by slow evaporation of acetone. Yield: 1.48 g (70%).

### S3. Refinement

All H-atoms were positioned geometrically and allowed to ride on their parent atoms, with C—H = 0.93 Å (aromatic), 0.96 Å (methyl group), with *U*_iso_(H) = 1.2 or 1.5*U*_eq_(C); for aromatic and methyl group.

### Crystal data

**Table:**

C_22_H_18_O	*Z* = 2
*M**_r_* = 298.36	*F*(000) = 316
Triclinic, *P*1	*D*_x_ = 1.224 Mg m^−^^3^
Hall symbol: -P 1	Melting point: 393 K
*a* = 7.6396 (3) Å	Mo *K*α radiation, λ = 0.71073 Å
*b* = 9.9106 (4) Å	Cell parameters from 7102 reflections
*c* = 11.9263 (4) Å	θ = 2.8–26.3°
α = 103.166 (2)°	µ = 0.07 mm^−^^1^
β = 104.713 (2)°	*T* = 273 K
γ = 103.308 (2)°	Block, colourless
*V* = 809.66 (6) Å^3^	0.40 × 0.35 × 0.30 mm

### Data collection

**Table:**

Bruker Kappa APEXII CCD diffractometer	4534 independent reflections
Radiation source: fine-focus sealed tube	3291 reflections with *I* > 2σ(*I*)
Graphite monochromator	*R*_int_ = 0.024
ω and φ scan	θ_max_ = 29.6°, θ_min_ = 2.2°
Absorption correction: multi-scan (*SADABS*; Bruker, 2004)	*h* = −10→10
*T*_min_ = 0.908, *T*_max_ = 1.000	*k* = −13→13
18636 measured reflections	*l* = −16→16

### Refinement

**Table:**

Refinement on *F*^2^	Primary atom site location: structure-invariant direct methods
Least-squares matrix: full	Secondary atom site location: difference Fourier map
*R*[*F*^2^ > 2σ(*F*^2^)] = 0.048	Hydrogen site location: inferred from neighbouring sites
*wR*(*F*^2^) = 0.151	H-atom parameters constrained
*S* = 1.06	*w* = 1/[σ^2^(*F*_o_^2^) + (0.073*P*)^2^ + 0.1155*P*] where *P* = (*F*_o_^2^ + 2*F*_c_^2^)/3
4534 reflections	(Δ/σ)_max_ = 0.001
209 parameters	Δρ_max_ = 0.23 e Å^−^^3^
0 restraints	Δρ_min_ = −0.17 e Å^−^^3^

### Special details

**Table:**

Geometry. Bond distances, angles *etc*. have been calculated using the rounded fractional coordinates. All su's are estimated from the variances of the (full) variance-covariance matrix. The cell e.s.d.'s are taken into account in the estimation of distances, angles and torsion angles

### Fractional atomic coordinates and isotropic or equivalent isotropic displacement parameters (Å^2^)

**Table:**

	*x*	*y*	*z*	*U*_iso_*/*U*_eq_	
O1	0.37261 (17)	0.60727 (11)	0.09681 (9)	0.0666 (4)	
C1	0.1424 (2)	0.25545 (16)	−0.31150 (12)	0.0598 (5)	
C2	0.02836 (17)	0.14897 (13)	−0.26587 (10)	0.0413 (3)	
C3	−0.08815 (19)	0.01392 (14)	−0.34508 (11)	0.0489 (4)	
C4	−0.1977 (2)	−0.08585 (15)	−0.30810 (13)	0.0555 (4)	
C5	−0.1963 (2)	−0.05199 (16)	−0.18929 (14)	0.0594 (5)	
C6	−0.0836 (2)	0.08192 (15)	−0.10901 (12)	0.0513 (4)	
C7	0.02996 (17)	0.18386 (13)	−0.14437 (10)	0.0396 (3)	
C8	0.14997 (19)	0.32479 (13)	−0.05697 (10)	0.0447 (3)	
C9	0.13129 (18)	0.38670 (14)	0.04796 (11)	0.0477 (4)	
C10	0.26312 (18)	0.52846 (14)	0.13063 (10)	0.0448 (4)	
C11	0.26370 (17)	0.57385 (13)	0.25891 (10)	0.0404 (3)	
C12	0.1919 (2)	0.47482 (14)	0.31431 (11)	0.0478 (4)	
C13	0.20814 (19)	0.51956 (14)	0.43612 (11)	0.0476 (4)	
C14	0.29252 (17)	0.66487 (13)	0.50571 (10)	0.0397 (3)	
C15	0.35955 (18)	0.76420 (13)	0.44861 (10)	0.0429 (3)	
C16	0.34808 (18)	0.71934 (13)	0.32782 (10)	0.0427 (3)	
C17	0.30998 (17)	0.71263 (14)	0.63651 (10)	0.0422 (3)	
C18	0.35892 (19)	0.62864 (15)	0.71095 (11)	0.0481 (4)	
C19	0.3769 (2)	0.67267 (17)	0.83319 (12)	0.0560 (5)	
C20	0.3468 (2)	0.80073 (19)	0.88272 (12)	0.0636 (5)	
C21	0.2975 (3)	0.8849 (2)	0.81035 (13)	0.0695 (6)	
C22	0.2805 (2)	0.84196 (17)	0.68813 (12)	0.0574 (5)	
H1A	0.12155	0.21253	−0.39637	0.0897*	
H1B	0.27494	0.28020	−0.26678	0.0897*	
H1C	0.10373	0.34179	−0.30056	0.0897*	
H3	−0.09204	−0.00968	−0.42594	0.0587*	
H4	−0.27269	−0.17613	−0.36309	0.0666*	
H5	−0.27074	−0.11885	−0.16353	0.0712*	
H6	−0.08350	0.10469	−0.02891	0.0615*	
H8	0.24937	0.37590	−0.07733	0.0536*	
H9	0.03187	0.33939	0.07040	0.0573*	
H12	0.13232	0.37751	0.26899	0.0574*	
H13	0.16190	0.45138	0.47211	0.0572*	
H15	0.41293	0.86238	0.49267	0.0514*	
H16	0.39719	0.78701	0.29226	0.0512*	
H18	0.37988	0.54158	0.67813	0.0576*	
H19	0.40953	0.61518	0.88177	0.0672*	
H20	0.35956	0.83064	0.96495	0.0763*	
H21	0.27551	0.97129	0.84371	0.0834*	
H22	0.24891	0.90049	0.64033	0.0688*	

### Atomic displacement parameters (Å^2^)

**Table:**

	*U*^11^	*U*^22^	*U*^33^	*U*^12^	*U*^13^	*U*^23^
O1	0.0822 (8)	0.0610 (6)	0.0424 (5)	−0.0025 (5)	0.0263 (5)	0.0071 (4)
C1	0.0701 (10)	0.0586 (8)	0.0426 (7)	0.0047 (7)	0.0249 (6)	0.0080 (6)
C2	0.0422 (6)	0.0426 (6)	0.0362 (5)	0.0136 (5)	0.0117 (4)	0.0071 (4)
C3	0.0517 (8)	0.0478 (7)	0.0379 (6)	0.0152 (6)	0.0086 (5)	0.0020 (5)
C4	0.0492 (8)	0.0431 (7)	0.0561 (8)	0.0065 (6)	0.0045 (6)	0.0030 (6)
C5	0.0536 (8)	0.0522 (8)	0.0661 (9)	0.0044 (6)	0.0186 (7)	0.0191 (7)
C6	0.0555 (8)	0.0524 (8)	0.0445 (6)	0.0119 (6)	0.0196 (6)	0.0130 (5)
C7	0.0407 (6)	0.0410 (6)	0.0352 (5)	0.0135 (5)	0.0112 (4)	0.0082 (4)
C8	0.0502 (7)	0.0439 (6)	0.0354 (5)	0.0097 (5)	0.0137 (5)	0.0084 (5)
C9	0.0459 (7)	0.0517 (7)	0.0371 (6)	0.0085 (6)	0.0139 (5)	0.0038 (5)
C10	0.0481 (7)	0.0484 (7)	0.0341 (5)	0.0138 (5)	0.0123 (5)	0.0075 (5)
C11	0.0410 (6)	0.0434 (6)	0.0329 (5)	0.0127 (5)	0.0100 (4)	0.0066 (4)
C12	0.0564 (8)	0.0383 (6)	0.0404 (6)	0.0067 (5)	0.0159 (5)	0.0040 (5)
C13	0.0565 (8)	0.0421 (7)	0.0408 (6)	0.0070 (6)	0.0183 (5)	0.0114 (5)
C14	0.0398 (6)	0.0430 (6)	0.0340 (5)	0.0128 (5)	0.0108 (4)	0.0083 (4)
C15	0.0497 (7)	0.0358 (6)	0.0359 (5)	0.0099 (5)	0.0094 (5)	0.0057 (4)
C16	0.0482 (7)	0.0406 (6)	0.0362 (5)	0.0100 (5)	0.0116 (5)	0.0120 (4)
C17	0.0395 (6)	0.0481 (7)	0.0347 (5)	0.0104 (5)	0.0121 (4)	0.0077 (5)
C18	0.0505 (7)	0.0489 (7)	0.0420 (6)	0.0085 (6)	0.0166 (5)	0.0136 (5)
C19	0.0536 (8)	0.0686 (9)	0.0429 (7)	0.0081 (7)	0.0165 (6)	0.0212 (6)
C20	0.0582 (9)	0.0913 (12)	0.0376 (6)	0.0204 (8)	0.0194 (6)	0.0108 (7)
C21	0.0794 (11)	0.0832 (11)	0.0489 (8)	0.0428 (9)	0.0238 (7)	0.0041 (7)
C22	0.0673 (9)	0.0667 (9)	0.0428 (7)	0.0353 (8)	0.0169 (6)	0.0116 (6)

### Geometric parameters (Å, º)

**Table:**

O1—C10	1.2192 (18)	C19—C20	1.369 (2)
C1—C2	1.499 (2)	C20—C21	1.375 (3)
C2—C3	1.3873 (18)	C21—C22	1.385 (2)
C2—C7	1.4072 (16)	C1—H1A	0.9600
C3—C4	1.370 (2)	C1—H1B	0.9600
C4—C5	1.376 (2)	C1—H1C	0.9600
C5—C6	1.377 (2)	C3—H3	0.9300
C6—C7	1.388 (2)	C4—H4	0.9300
C7—C8	1.4628 (17)	C5—H5	0.9300
C8—C9	1.3207 (17)	C6—H6	0.9300
C9—C10	1.4743 (19)	C8—H8	0.9300
C10—C11	1.4917 (16)	C9—H9	0.9300
C11—C12	1.3901 (19)	C12—H12	0.9300
C11—C16	1.3904 (18)	C13—H13	0.9300
C12—C13	1.3828 (17)	C15—H15	0.9300
C13—C14	1.3909 (18)	C16—H16	0.9300
C14—C15	1.3948 (18)	C18—H18	0.9300
C14—C17	1.4847 (16)	C19—H19	0.9300
C15—C16	1.3809 (16)	C20—H20	0.9300
C17—C18	1.3900 (19)	C21—H21	0.9300
C17—C22	1.384 (2)	C22—H22	0.9300
C18—C19	1.3840 (18)		
			
C1—C2—C3	120.06 (11)	C2—C1—H1C	109.00
C1—C2—C7	121.73 (11)	H1A—C1—H1B	109.00
C3—C2—C7	118.16 (12)	H1A—C1—H1C	109.00
C2—C3—C4	122.16 (12)	H1B—C1—H1C	109.00
C3—C4—C5	119.78 (14)	C2—C3—H3	119.00
C4—C5—C6	119.30 (15)	C4—C3—H3	119.00
C5—C6—C7	121.79 (13)	C3—C4—H4	120.00
C2—C7—C6	118.80 (12)	C5—C4—H4	120.00
C2—C7—C8	120.48 (12)	C4—C5—H5	120.00
C6—C7—C8	120.72 (11)	C6—C5—H5	120.00
C7—C8—C9	126.52 (13)	C5—C6—H6	119.00
C8—C9—C10	122.06 (13)	C7—C6—H6	119.00
O1—C10—C9	121.45 (11)	C7—C8—H8	117.00
O1—C10—C11	119.84 (12)	C9—C8—H8	117.00
C9—C10—C11	118.70 (12)	C8—C9—H9	119.00
C10—C11—C12	122.41 (11)	C10—C9—H9	119.00
C10—C11—C16	118.91 (11)	C11—C12—H12	120.00
C12—C11—C16	118.59 (11)	C13—C12—H12	120.00
C11—C12—C13	120.66 (12)	C12—C13—H13	119.00
C12—C13—C14	121.08 (13)	C14—C13—H13	119.00
C13—C14—C15	117.89 (11)	C14—C15—H15	119.00
C13—C14—C17	120.99 (12)	C16—C15—H15	119.00
C15—C14—C17	121.12 (11)	C11—C16—H16	120.00
C14—C15—C16	121.17 (12)	C15—C16—H16	120.00
C11—C16—C15	120.55 (12)	C17—C18—H18	120.00
C14—C17—C18	120.50 (12)	C19—C18—H18	120.00
C14—C17—C22	121.33 (12)	C18—C19—H19	120.00
C18—C17—C22	118.16 (11)	C20—C19—H19	120.00
C17—C18—C19	120.97 (14)	C19—C20—H20	120.00
C18—C19—C20	120.12 (14)	C21—C20—H20	120.00
C19—C20—C21	119.72 (13)	C20—C21—H21	120.00
C20—C21—C22	120.46 (17)	C22—C21—H21	120.00
C17—C22—C21	120.57 (15)	C17—C22—H22	120.00
C2—C1—H1A	109.00	C21—C22—H22	120.00
C2—C1—H1B	109.00		
			
C1—C2—C3—C4	−178.74 (14)	C16—C11—C12—C13	−1.5 (2)
C7—C2—C3—C4	−1.1 (2)	C10—C11—C16—C15	−176.84 (13)
C1—C2—C7—C6	177.97 (13)	C12—C11—C16—C15	−0.2 (2)
C1—C2—C7—C8	−2.3 (2)	C11—C12—C13—C14	1.5 (2)
C3—C2—C7—C6	0.4 (2)	C12—C13—C14—C15	0.3 (2)
C3—C2—C7—C8	−179.93 (14)	C12—C13—C14—C17	−179.94 (14)
C2—C3—C4—C5	1.1 (2)	C13—C14—C15—C16	−2.0 (2)
C3—C4—C5—C6	−0.3 (2)	C17—C14—C15—C16	178.22 (13)
C4—C5—C6—C7	−0.4 (2)	C13—C14—C17—C18	40.6 (2)
C5—C6—C7—C2	0.4 (2)	C13—C14—C17—C22	−140.18 (15)
C5—C6—C7—C8	−179.34 (14)	C15—C14—C17—C18	−139.63 (15)
C2—C7—C8—C9	161.41 (14)	C15—C14—C17—C22	39.6 (2)
C6—C7—C8—C9	−18.9 (2)	C14—C15—C16—C11	2.0 (2)
C7—C8—C9—C10	178.77 (13)	C14—C17—C18—C19	179.54 (14)
C8—C9—C10—O1	13.9 (2)	C22—C17—C18—C19	0.3 (2)
C8—C9—C10—C11	−164.91 (13)	C14—C17—C22—C21	−179.96 (16)
O1—C10—C11—C12	−158.73 (15)	C18—C17—C22—C21	−0.7 (2)
O1—C10—C11—C16	17.8 (2)	C17—C18—C19—C20	−0.1 (2)
C9—C10—C11—C12	20.1 (2)	C18—C19—C20—C21	0.4 (3)
C9—C10—C11—C16	−163.39 (13)	C19—C20—C21—C22	−0.8 (3)
C10—C11—C12—C13	175.01 (14)	C20—C21—C22—C17	1.0 (3)

### Hydrogen-bond geometry (Å, º)

Cg1, Cg2 and Cg3 are the centroids of the C2–C7 methylbenzene, C11–C16 benzene and C17–C22 phenyl rings, respectively.

**Table:**

*D*—H···*A*	*D*—H	H···*A*	*D*···*A*	*D*—H···*A*
C1—H1*C*···*Cg*2^i^	0.96	2.97	3.6689 (17)	131
C5—H5···*Cg*3^ii^	0.93	2.84	3.5126 (18)	130
C21—H21···*Cg*1^iii^	0.93	2.99	3.632 (2)	127

Symmetry codes: (i) −*x*, −*y*+1, −*z*; (ii) *x*−1, *y*−1, *z*−1; (iii) *x*, *y*+1, *z*+1.
